# Habitat complexity and predator odours impact on the stress response and antipredation behaviour in coral reef fish

**DOI:** 10.1371/journal.pone.0286570

**Published:** 2023-06-28

**Authors:** Eric P. Fakan, Bridie J. M. Allan, Björn Illing, Andrew S. Hoey, Mark I. McCormick

**Affiliations:** 1 ARC Centre of Excellence for Coral Reef Studies, James Cook University, Townsville, QLD, Australia; 2 College of Sciences and Engineering, James Cook University, Townsville, QLD, Australia; 3 Department of Marine Science, University of Otago, Dunedin, New Zealand; 4 Thünen Institute of Fisheries Ecology, Bremerhaven, Germany; 5 Coastal Marine Field Station, School of Science, University of Waikato, Tauranga, New Zealand; University of Windsor, CANADA

## Abstract

Mass coral bleaching events coupled with local stressors have caused regional-scale loss of corals on reefs globally. Following the loss of corals, the structural complexity of these habitats is often reduced. By providing shelter, obscuring visual information, or physically impeding predators, habitat complexity can influence predation risk and the perception of risk by prey. Yet little is known on how habitat complexity and risk assessment interact to influence predator-prey interactions. To better understand how prey’s perception of threats may shift in degraded ecosystems, we reared juvenile *Pomacentrus chrysurus* in environments of various habitat complexity levels and then exposed them to olfactory risk odours before simulating a predator strike. We found that the fast-start escape responses were enhanced when forewarned with olfactory cues of a predator and in environments of increasing complexity. However, no interaction between complexity and olfactory cues was observed in escape responses. To ascertain if the mechanisms used to modify these escape responses were facilitated through hormonal pathways, we conducted whole-body cortisol analysis. Cortisol concentrations interacted with habitat complexity and risk odours, such that *P*. *chrysurus* exhibited elevated cortisol levels when forewarned with predator odours, but only when complexity levels were low. Our study suggests that as complexity is lost, prey may more appropriately assess predation risk, likely as a result of receiving additional visual information. Prey’s ability to modify their responses depending on the environmental context suggests that they may be able to partly alleviate the risk of increased predator-prey interactions as structural complexity is reduced.

## Introduction

Globally, ecosystems are faced with a variety of climate-induced and local anthropogenic stressors that lead to degraded systems with reduced structural complexity and lower species diversity [1,2]. Coral reefs are particularly vulnerable to such degradation, largely due to the thermal sensitivities of the corals themselves [3]. As the frequency and severity of marine heatwaves is increasing worldwide, coral bleaching and subsequent mortality is predicted to intensify [4], likely leading to further reductions in habitat complexity [5]. Reduction in the structural complexity of reef habitats has considerable implications for coral reef fishes, which are often closely tied to the physical characteristics of their habitat [6]. For many reef fishes, structurally complex branching corals represent preferred settlement habitats due to the provision of refugia, and the loss of these corals leads to reduced abundances and diversity of reef fish assemblages as exposure to predators and predation risk increases [7]. As many coral reef habitats are in a state of change, it is important to understand how structural complexity interacts with the perception of risk in reef fishes.

Habitat complexity can influence predator-prey interactions through the provision of shelter and by altering visibility, detection, and responses of both prey and predators, and thereby the probability of a predation event to occur and its success [8]. Habitat complexity is therefore an important driver of the spatial variation in predation risk [9]. The way prey perceive their environment is in part controlled by the fear of predation [10], and the spatial variation in predation risk throughout their environment i.e., ‘landscape of fear’, which describes the trade-off prey encounter across a risk gradient [11,12]. For example, following the reintroduction of wolves (*Canis lupus*) to the Yellowstone National Park (USA), the spatial distribution of elk (*Cervus elaphus*) increased in structurally more complex woodlands, highlighting that elk assessed open grasslands to be more dangerous when wolves were present [13]. This study emphasizes that structural complexity is an important component of both a prey’s perceived and actual risk of predation within its environment.

While a lack of, or lagged response, to a predator may be costly in terms of survival, continually responding to non-lethal threats is energetically costly as prey forego foraging and social opportunities [14,15]; therefore, the first step in avoiding predation is assessing the risk of predation. However, prey usually have incomplete information about their surrounding environment resulting in less than accurate assessments [16], forcing prey to over- or under-estimate risk [17]. The more information a prey has about predation threats, the better it will be able to optimize the balance between vigilance and other fitness-related behaviours. Consequently, prey often use multiple cues to identify potential predators, assess the level of risk they pose, and modify anti-predation behaviour appropriately [18–20].

Combining information from multiple cues can provide a more accurate assessment of risk, promoting optimal responses [21]. In many fishes the main senses used to assess risk are vision, olfaction, and mechanoreception (e.g. hearing, vibrations) [22–24]. Using olfactory cues alone, prey can quickly detect predator odours but these cues may linger after a predator has departed the area, leading to overestimations of risk [25]. In contrast, visual cues can provide immediate information on predator size, location and motivation, but prey may be more exposed to predation risk in obtaining visual cues [17,26]. When used in conjunction, odours and visual cues aid in positively identifying threats and determining the motivation of potential predators, and are therefore, important components within the decision matrix whereby prey determine the appropriate response [18,27].

If predator avoidance is unsuccessful and a potential prey is exposed to a predator, prey may reduce predation risk through behavioural responses such as maintaining a safe distance, decreasing activity, increased vigilance and/or initiating an escape response [28,29]. In fishes, a fast-start escape response is a common anti-predation behavior, which involves a short but high energy swimming burst typically in a C-shaped motion [29,30]. Faster escape responses have been found to increase the likelihood of prey escaping a predator [31] and be good predictors of survivorship of fishes in the wild [32]. Fishes can optimize their escape response to a predatory threat depending upon the information available, including the presence and motivation of a predator [33–36]. In particular, the presence of chemical alarm cues, such as odours from the damaged skin of conspecifics, prior to a predatory strike has been found to ‘forewarn’ prey, leading to more effective escape responses [36,37].

This optimized escape response through forewarning may have at its basis a cortisol stress response [38,39] as the synthesis of cortisol can prime decisions and movements [40]. As such, it has been hypothesized that increased cortisol synthesis in response to predators and/or cues may regulate the sensory processing used in detecting predators and ultimately shape prey’s responses [9,39,41]. The mechanism that facilitates this improved antipredator response may be associated with the stress response [42,43]. In vertebrates, the primary response to stress is a rapid elevation in glucocorticoids (such as cortisol or corticosterone), which quickly releases glucose into the blood priming the body for increased activity if required (secondary stress response) [43]. As such, olfactory cues of risk have been shown to alter the behaviour and physiology in fishes [44,45]. For instance, risk cues have been shown to increase cortisol concentrations and induce defensive behaviours in zebrafish (*Danio rerio*) [46], likely in anticipation of an imminent threat. To some extent, the stress response may have a positive effect on prey by improving reaction times to a strike, and hence their probability of escape.

It is expected that the degradation of coral reefs will influence how predators interact with their prey and the relative balance of senses prey use to judge risk during predator-prey interactions [47]. As habitat complexity decreases, the visibility and olfactory cues of prey to predators (and vice versa) will increase [48,49], potentially making degraded environments perceived as riskier for prey. While it is known that risk cues can cause an increase in cortisol levels [44,46], habitat complexity levels have resulted in various impacts on cortisol levels; with reduced complexity decreasing [50], increasing [51,52], or having no impact [53,54] on cortisol concentrations. A better understanding of the interaction between habitat complexity and risk assessment is required to interpret how the dynamics of predator-prey interactions may change as an ecosystems degrades [55].

The aim of this study was to examine how levels of habitat complexity interact with olfactory predator cues in modifying the fast-start escape response of a common coral reef damselfish, *Pomacentrus chrysurus*, and whether the mechanism underlying the response was cortisol-related. Newly-settled damselfish were chosen as prey because the transition from pelagic larvae to settled juveniles represents a critical bottleneck where mortality is extreme [56,57]. Newly-settled *P*. *chrysurus* were reared in tanks containing one of three levels of topographic complexity for two weeks and then their fast-start responses were measured in the presence or absence of odours from a known predator. We predicted that predator odours would heighten the escape response of *P*. *chrysurus* and that lower levels of complexity would further exacerbate this response. Specifically, we predicted that prey fish would recognize predator odours as a threat and enhance their fast-start response. Prey may associate a lower complexity environment with higher risk and therefore, be more vigilant and respond sooner and more strongly to any perceived threat. Additionally, we hypothesized that if altered, escape responses induced by predator odours and/or habitat complexity may be mediated through elevated cortisol concentrations.

## Methods

### Study species & housing conditions

*Pomacentrus chrysurus*, the whitetail damselfish is a rubble-associated, omnivorous fish that is common across the Indo-Pacific. Recent research has shown that the whitetail damselfish’s ability to learn and respond to predators can be impacted by coral degradation [58]. Fish were collected around Lizard Island (14°40’S, 145°28’E), northern Great Barrier Reef (GBR) in November of 2016. Naïve settlement-stage whitetail damselfish that had not being exposed to reef-based predators were collected with light traps moored at least 50 m off the fringing reef. Light traps were deployed at dusk and collected at dawn the following morning and the catch was transported to the Lizard Island Research Stations aquarium facility in 68 L tanks. The catch was immediately sorted and all whitetail damselfish captured were haphazardly placed into 32 L plastic tanks (432 x 324 x 305mm) containing one of three levels of complexity for 2 weeks prior to the start of the experiment. Three replicate tanks of each complexity were established with ~45 fish per tank. The three levels of complexity were manipulated by altering the number of resin coral models (~8 × 3 × 5 cm) within the tanks: no corals (low complexity), 3 model corals (medium complexity), and 6 model corals (high complexity). The structural complexity or rearing environments can play an important role in shaping the behavioral phenotypes in captive fishes [59,60]. The standard length (SL) of the whitetail damselfish (16.2 ± 0.7 mm) was measured following the 2-week housing, from kinematic videos. Fish were fed *ad libitum* with newly hatched *Artemia* twice daily, but not fed 12 h prior to commencement of the experimental trials to standardise for satiation in the trials.

The piscivorous rock cod, *Cephalopholis boenak*, was selected as a model predator as it is known to prey on juvenile damselfishes [61]. Two *C*. *boenak*, were caught using hand nets and an anaesthetic clove oil solution and transferred immediately to individual 68 L aquaria with 5–7 PVC tubes as shelter where they were housed until they were used in the trials. Each aquaria was supplied with fresh flow-through seawater and supplemental aeration. *C*. *boenak* were fed juvenile cardinalfish daily (Apogonidae: *Cheilodipterus spp*.), which is phylogenetically distant from the target damselfish to avoid providing information through diet [62]. Newly recruited cardinalfish were caught in light traps and housed in 32L plastic tanks with fresh flow-through seawater and fed as per whitetail damselfishes. Every day, one cardinalfish per *C*. *boenak* were euthanised through cold shock using an ice slurry, and a net was used to ensure fish did not directly contact ice. This method of euthanasia has been shown to be fast (~7 s) and cause little distress in small fishes [63]. The herbivorous surgeonfish, *Acanthurus nigrofuscus*, were used as a behavioural control for olfactory cues of any non-threating heterospecific fish species. Two *A*. *nigrofuscus* were caught using a barrier net and hand nets, and transferred immediately to individual 68L aquaria supplied with fresh flow-through seawater, supplemental aeration and 5–7 PVC tubes as shelter. *A*. *nigrofuscus* were regularly provided with fresh pieces of coral rubble covered in algal turfs on which to graze. All fish were transported to the Lizard Island Research Stations aquarium facility in 68 L tanks within an hour of capture and no mortality occurred during transportation or housing periods. Following experimental trials, surgeonfish and rock cods were released near the original capture site, and the remaining damselfishes and cardinalfishes were distributed over multiple reefs.

### Animal ethics

Research was carried out under approval of the James Cook University animal ethics committee (permit: A2005, A2080) and according to the University’s animal ethics guidelines.

### Experimental overview

**Fig 1:**
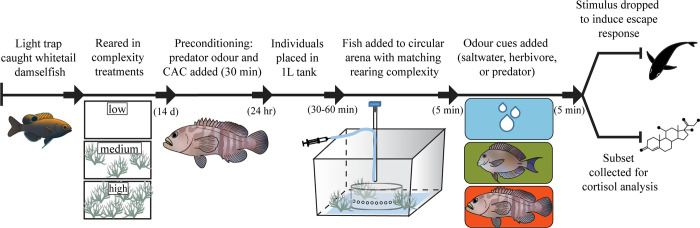
Schematic diagram of the timeline for treatment conditioning and experimental design. On the 15^th^ day in complexity treatments all juvenile *P*. *chrysurus* were trained to associate predator odours (*C*. *boenak*) as a threat with chemical alarm cues (CAC). After acclimation in circular arena, either saltwater, herbivore (*A*. *nigrofuscus*) or predator odour was introduced for 5 minutes. Most fish were startled by a stimulus to induce fast-start escape response, which were recorded from below, while a subset of saltwater and predator samples were collected for cortisol analysis. See S1 Fig for more details on experimental tank.

In summary, settlement-stage *P*. *chrysurus* were reared within tanks that had one of three levels of habitat complexity (low, medium, or high) for 15 days. All fish were conditioned to recognize predator odours as a threat (see pre-conditioning below for details). Following rearing, individual *P*. *chrysurus* were placed into a fast-start escape trial tank with complexity that matched the rearing environments and exposed (5 min) to one of three olfactory cues (predator odour, herbivore odour, or a saltwater control). A weighted stimulus was then dropped from overhead to elicit a fast-start escape response, which was recorded at high speed for kinematic analysis. A subset of fish were collected following the odour acclimation, but prior to the stimulus dropping, to quantify cortisol concentrations. See Fig 1 for an overview of the timeline and experimental design. Odours from the piscivore, *C*. *boenak*, were used to represent a threat while odours from the herbivore, *A*. *nigrofuscus*, were used as a behavioural control to account for the effect of exposing *P*. *chrysurus* to olfactory cues of any non-threating heterospecific fish species. The saltwater treatment was used to control for the injection of cues.

### Pre-conditioning

Fifteen days after being placed into one of three topographic complexity treatments (see above), fish were pre-conditioned to associate the smell of *C*. *boenak* as a threat using associative learning. This was done to ensure that the experimental whitetail damselfish associated *C*. *boenak* as a threat because these fish were naïve to reef-based predators. Pre-conditioning occurred only once and was conducted the day prior to experimental trails. This involved pairing alarm cues released by damaged juvenile whitetail damselfish with the odour of the predator. Chemical alarm cues (CAC) were obtained through 5 superficial cuts to both sides of 4 euthanised (as described above) whitetail damselfish and each rinsed with 15 ml of seawater [64]. The odour from the predator was prepared by turning off the flowing seawater and leaving the tank containing the predators with aeration for 3 h. Prey were exposed to 1 L of water from the predator tank (to their 32 L tank) plus 60 ml of CAC for 30 min [65]. These were injected simultaneously into the tanks housing the *P*. *chrysurus* to be used in all experimental trials for the day, which was left without water flow (just aeration) for 30 min. This coupling of the predator odour cues with a CAC leads to the assignment of risk to the cues through a process known as associative learning. Previous research has shown that newly settled damselfish can learn to associate the olfactory cue of a predator with risk after only one exposure when paired with a CAC [66].

### Experimental protocol

Escape responses were examined in a transparent circular acrylic arena (diameter 200mm; height 100 mm), within a large opaque-sided plastic tank (585 length x 420 width x 330 mm height; 60 L) with a transparent Perspex bottom to allow responses to be filmed as a silhouette from below. The circular arena was used to ensure that the focal individual remained roughly in the center of the tank where the stimulus was dropped. The large tank was illuminated by a LED light strip wrapped around the outside of the tank with light penetrating with even illumination through the opaque sides. The water level was maintained at 80 mm (~ 20 L) to reduce movements in the vertical plane, and the water in the tank was emptied and refilled with flow-through seawater after every trial to avoid a build-up of odours and to maintain temperature. The same plastic corals used in the rearing tanks were randomly placed within the experimental tank (excluding arena), representing various complexity levels each individual were reared in. See S1 Fig for more details on experimental tank.

At the beginning of each trial, fish were caught using a fine-mesh handnet and transferred to a 1 L acclimation tank (one fish/tank) held within a water bath (to maintain the same temperature) and left for 30–60 min. Fish were then carefully poured individually to the arena and allowed to acclimate to the tank and complexity level for 5 min. The acclimation stage in the 1 L tank reduced the time that fish took to begin exploring the arena, suggesting it made the transfer process less disturbing. The complexity level in each trial matched the individuals’ rearing complexity level, with complexity manipulated using coral models placed around but not inside the fast-start arena. Odours were then introduced into the arena through a clear plastic tube attached to the stimulus weight tube that hung over the center of the arena and fish were left for an additional 5 min. It has been previously shown that 5 minutes is sufficient to induce an acute stress response, measured as cortisol, in marine fishes [67]. Holes in the arena allowed flow of water during acclimation. Predator and herbivore odour treatments involved the slow injection of 15 ml of seawater from either the *C*. *boenak* or *A*. *nigrofuscus* holding tanks (prepared as described above) and flushed with seawater (15 ml) to ensure all the odours entered the arena. The saltwater control consisted of slowly injecting 30 ml of seawater. All odours introduced within the escape trials excluded CAC. All olfactory injection tubes were flushed with 60 ml of seawater between trials. After the second 5 min acclimation within the arena, fish were startled with the release of the stimulus weight, which was suspended within a 48.5 mm diameter white PVC pipe placed directly over the center of the arena. The 48.5 mm diameter PVC pipe was used as a reference scale for calibration. The stimulus was released into the water using an electromagnet and remained invisible to the juvenile fish until the falling stimulus touched the water surface. The stimulus was only released once the fish swam into the central region of the tank which allowed all individuals to move an equal distance in any direction and standardized for fish position relative to the stimulus. A monofilament line, attached to the stimulus weight, prevented the stimulus from hitting the bottom of the arena, thus ensuring the escape response was triggered by the stimulus breaching the water surface. To ensure a standardized protocol, prey escape variables were only measured when prey performed a C-start (commencement of fast-start that results in the individual forming a C-shape). High-speed (480 fps) videos of fast starts were filmed using a Casio ZR1000 camera. See S1 Fig for visualization and description of kinematic trial setup. All trials were conducted between 10:00 and 17:00 h. Salinity (35 ppt) and temperature (29˚C) were kept constant throughout the study period and trials. The sample sizes for low complexity trials were n = 20, 20, and 19 for control, herbivore and predator, respectively. For medium complexity trials n = 20, 20, and 21 and lastly for high complexity trials n = 20, 20, and 23 (control, herbivore, predator), respectively.

To determine if the predator odor cue caused a physiological stress response, a subsample of fish were collected for whole-body cortisol analysis via enzyme-linked immunosorbent assay (ELISA; details below). These fish were subjected to the exact same protocol up until when they were startled with the stimulus (see above). Just prior to the startle but following the 5 min acclimation to odour treatments, fish were quickly removed from the arena, rapidly killed with cold shock (as described above) and stored in liquid nitrogen for subsequent cortisol analysis. The herbivore treatment was used as a behavioural control and therefore no herbivore samples were undertaken for cortisol analysis. Sample sizes for cortisol from low complexity trials were n = 10 and 12 (control and predator respectively) and for medium complexity trials n = 9 and 11 and high complexity trials n = 9 and 11.

### Kinematic analysis

Kinematic variables associated with the fast-start escape responses were analysed using the image-analysis software Image-J, with the manual tracking plug-in (imagej.nih.gov/ij/). The centre of mass of each fish was tracked through stage 1 and 2, (i.e., the first two axial bends, defined based on Domenici and Blake [30]), which is the period considered crucial for avoiding predator attacks. The following kinematic variables were measured:

1. Response latency (s) was measured as the time interval between the stimulus onset and the first detectable movement leading to the escape of the animal.
2. Response speed (m s^-1^) was measured as the distance covered within a fixed time (41 ms). This fixed duration was based on the average duration of stage 1 and 2 from juveniles of a similar species [68].
3. Maximum response speed (m s^-1^) was measured as the maximum speed achieved during any frame during stage 1 and stage 2 [30].
4. Maximum acceleration (m s^-2^) was measured as the maximum increase in speed between frames during stage 1 and stage 2.
5. Response distance (m) is a measure of the total distance covered by the fish during stage 1 and 2.

### Cortisol extraction and ELISA validations

Whole-body cortisol was extracted using a method described by Allan et al., [69] and was measured with a commercially available cortisol ELISA kit (Cayman Chemical Item Number 500360), in March of 2019. Briefly, individual fish were freeze-dried (Christ Alpha 1–2 LDplus, 0.2 mbar, >16 h) and weighed (Mettler Toledo UMX2 Ultra-Microbalance, 0.1 μg readability) prior to being homogenized in 2 ml Eppendorf vials, using a glass bead and 0.5 ml phosphate-buffered saline (PBS, pH 7.4) in a shaking mill (MP Biomedical FastPrep24) for 3 min. The homogenate was then transferred to a 10 ml glass vial and rinsed with an additional 0.4 ml of PBS and then ethyl acetate was added at a 1:9 ratio. The samples were vortexed for 1 min before being centrifuged (Eppendorf 5810 R) at 3,500 rpm for 5 min at 4°C. The supernatant was collected, and the extraction steps were performed four times, pooling each extraction step. The ethyl acetate was dried off in a centrifugal vacuum concentrator (Thermo Savant SpeedVac SC110A, 43°C) and the samples were reconstituted within 48 hours using 1 ml assay buffer. The samples were analysed in triplicates with a spectrophotometer (SpectraMax Plus 384 Microplate Reader, Molecular Devices) and the average absorbance was calculated from readings between 405 and 420 nm. See Allan et al., [69] for more details on cortisol extraction.

Prior to measuring cortisol concentrations, assay validations steps (parallelism, accuracy and precision) were performed for the cortisol ELISA kit, following recommendations by Metcalfe et al., [70]. Parallelism was confirmed by an ANCOVA’s homogeneity of slopes assumption by comparing dose–response curves of diluted samples for each fish against a standard curve (ANCOVA, P>0.05, n = 3; S2 Fig). In brief, the reconstituted samples (n = 3) were diluted (1:1, 1:2, 1:4, 1:8, 1:16, 1:32, 1:64, and 1:128) and compared against the cortisol standard curve (Cayman Chemical ELISA kit, 6.6–4000 pg ml^-1^ range). The optimal dilution for the samples fell between the dilutions 1:8 and 1:16; therefore, a sample dilution of 1:12 was chosen to achieve a 50% relative maximum binding and only sample dilutions falling within 20–80% B/B_0_ relative maximum binding were accepted. The accuracy or recovery rate of the extraction method was assessed by spiking samples (n = 4) with 800 pg cortisol ml^-1^. For each of the four samples, two fish were homogenized, pooled and split into halves, with one half receiving the spike and the other the assay buffer. Both halves were then processed in the same way as all other samples. The spike’s recovery (percentage) was expressed as spiked sample result−unspiked sample result×100/known spike (800 pg ml^-1^), and the mean recovery (78.5%, n = 4) was used as a correction factor for calculating the samples’ cortisol concentration. Intra-assay precision of triplicate samples was determined using the coefficient of variation (CV) and found to be 3.8±3.6 (mean±s.d., n = 62). See S2 Fig for dose–response curves, comparison between standard curve and recovery rate from method of extraction.

### Statistical analyses

#### Escape response

Of our 183 kinematic trials, 143 (78.1% across all treatments) performed C-start escape responses. To investigate if the frequency of responsiveness [29] was impacted by treatments, a Pearson’s Chi-square test was used and found no association between performing escape responses and treatments (χ^2^_8_ = 12.13, p = 0.146; See S1 Table for sample sizes). A similar level of responsiveness of *P*. *chrysurus* to burst stimulus has been shown in other studies [71]. The latency to respond to the stimulus was positively related to distance to the stimulus (ANCOVA: F_1,133_ = 77.09, p<0.001) and the nature of the relationship was consistent among treatments (homogeneous slopes: F_4,125_ = 0.64, p = 0.637). Therefore, the residuals of this relationship were used in subsequent analyses to remove the influence of the distance to the stimulus on latency alone. No other variables were impacted by distance to the stimulus. A two-way multivariate analysis of covariance (MANCOVA) was performed to test whether there were differences in the escape response variables among complexity treatments (low, medium, and high), potential forewarning odours (predator, herbivore, and saltwater control), and their interaction. Complexity and odour treatments were fixed factors, the five escape variables were used as dependent variables, and the standard length of the fish was used as a covariate. Box’s M test was satisfied (P = 0.095). All dependent variables from the MANCOVA were further examined using a two-way analysis of covariance (ANCOVA) with Bonferroni-corrected comparison post-hoc tests to determine the nature of the significant difference found within the ANCOVA’s. Post-hoc comparisons from significant ANCOVA’s are provided in supplemental materials (see S2 Table). Partial eta-squared (η^2^) are given as an estimate of effect size. The assumption of normality was visually examined (Q-Q plots) and homogeneity of variance tested (Levene’s test). Response and kinematic analyses were performed in SPSS (IBM, version 27).

#### Physiological stress response

A two-way ANOVA was used to examine the effect of complexity (low, medium, high) and odours (control, predator) on whole-body cortisol concentrations. Tukey’s HSD post-hoc tests were conducted to determine the nature of any significant differences from the ANOVA. Post-hoc comparisons are provided in supplemental materials (see S3 Table). Partial eta-squared are given as an estimate of effect size. The assumption of normality was visually examined (Q-Q plots) and homogeneity of variance tested (Levene’s test). Cortisol analysis was performed in R, version 3.5.1 [72].

### Results

#### Escape response

**Fig 2:**
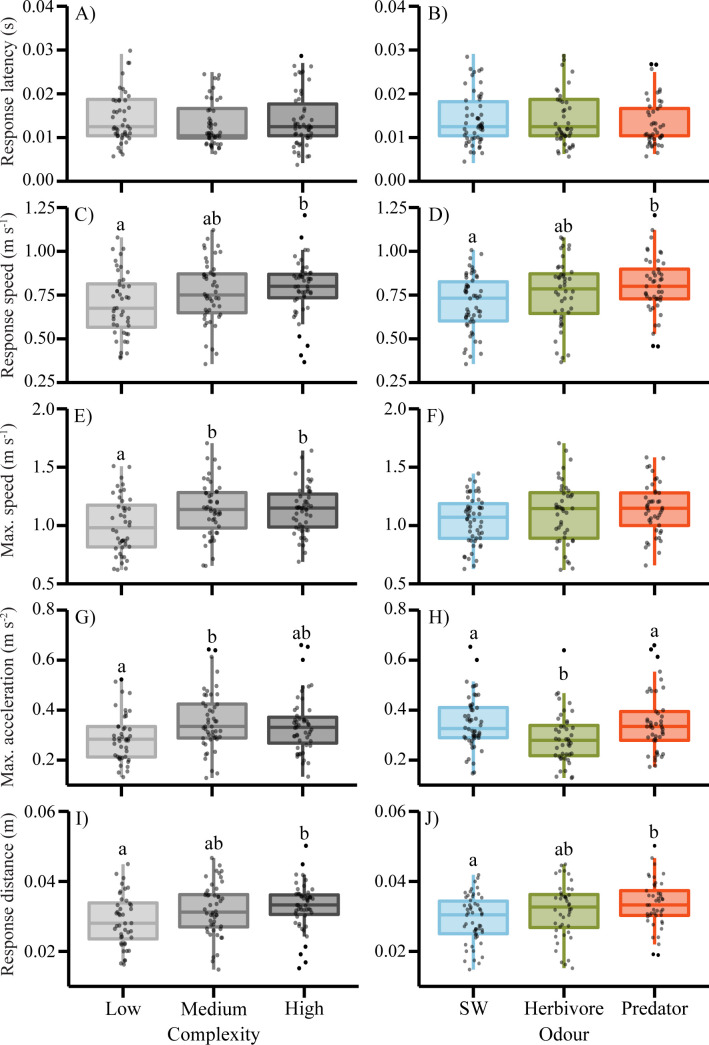
Effect of complexity and forewarning odours on escape response and kinematic of *Pomacentrus chrysurus*. Boxplots show the median and 25% quantiles, black dots are outliers, grey dots are raw data points for response latency (A,B), response speed (C,D), maximum speed (E,F), maximum acceleration (H,I), and response distance (J,K). Letters above bars represent LSD groupings of means. Sample sizes for complexity trials were n = 44, 48, and 51 (low, medium, high), and for predator odour trials n = 54, 44, and 45 (control, herbivore, predator), respectively.

A two-way MANCOVA revealed a significant difference in the escape performance based on complexity (Pillai’s trace 0.14, F_10, 260_ = 1.89, p = 0.047) and odour (Pillai’s trace 0.20, F_10, 260_ = 2.85, p = 0.002), but no interaction effect was observed (Pillai’s trace 0.19, F_20, 528_ = 1.28, p = 0.188). The nature of these differences was further explored by two-way ANCOVAs. These indicated that nearly all fast-start variables were impacted by the level of complexity and the forewarning odour, however, no interaction effects were observed (Table 1). High complexity resulted in faster response speed (Fig 2; F_2,133_ = 3.83, p = 0.024), higher maximum speed (F_2,133_ = 5.62, p = 0.005) and an increase in response distance (F_2,133_ = 3.83, p = 0.024), when compared to low levels of complexity. Compared to the saltwater controls, exposure to predator odour significantly increased the response speed (F_2,133_ = 4.45, p = 0.013), and escape distance (F_2,133_ = 4.45, p = 0.013), while exposure to herbivore odour reduced maximum acceleration (F_2,133_ = 4.77, p = 0.010). Neither complexity levels nor forewarning odours were found to have an impact on response latency (F_2,133_ = 0.74, p = 0.481; F_2,133_ = 1.22, p = 0.299, respectively).

**Table 1:** Results of two-way ANCOVA’s on the fast-start response variables for *Pomacentrus chrysurus*.

	Complexity _(2,133)_			Odour _(2,133)_			Complexity*Odour _(4,133)_		
Variable	F	p	η^2^	F	p	η^2^	F	p	η^2^
Latency	0.74	0.481	0.011	1.22	0.299	0.018	1.37	0.249	0.039
Speed	3.83	**0.024**	0.054	4.45	**0.013**	0.063	1.19	0.318	0.035
Max Speed	5.62	**0.005**	0.078	2.15	0.121	0.031	1.55	0.191	0.045
Max Accel	5.19	**0.007**	0.072	4.77	**0.010**	0.067	0.85	0.495	0.025
Distance	3.83	**0.024**	0.054	4.45	**0.013**	0.063	1.19	0.318	0.035

Summary of the fast-start escape response variables: Response latency (s), speed (m s^-1^), maximum speed (m s^-1^), maximum acceleration (m s^-2^), and response distance (m) in juvenile *P*. *chrysurus* reared with varying levels of complexity (low, medium and high) and presented with potential forewarning odours (predator, herbivore, and salt water). Standard length was used as a covariate. Degree of freedom are presented with each model and bold values are significant at alpha = 0.05. Partial eta-squared (η^2^) are given as an estimate of effect size.

### Physiological stress response

**Fig 3:**
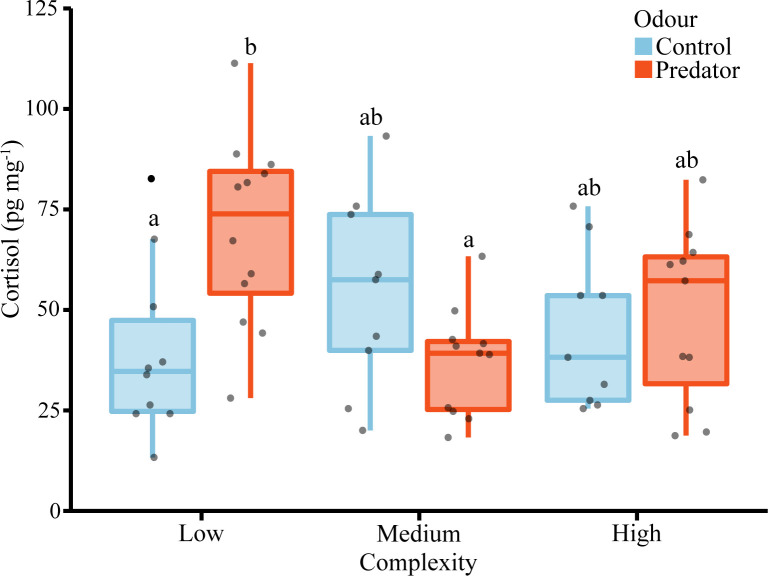
Effect of complexity and forewarning odours on cortisol concentrations of juvenile *Pomacentrus chrysurus*. Boxplots show the median and 25% quantiles, black dots are outliers, grey dots are raw data points and letters above bars represent Tukey’s HSD groupings of means. Sample sizes for control odours were n = 10, 9, and 9 (low, medium, high) and for predator odours n = 12, 11, and 11 (low, medium, high), respectively.

A two-way ANOVA revealed a significant interaction between complexity and odour on the concentration of cortisol (Table 2: F_2,56_ = 6.66, p = 0.003). Juvenile *P*. *chrysurus* reared in the low complexity treatment and exposed to predator odours prior to the burst stimulus had significantly higher cortisol levels than fish from low complexity saltwater controls and from the medium complexity predator odour treatment (Fig 3). In the low complexity treatments, the mean (± SE) cortisol concentrations of fish treated with sea water and predator odour water were 39.56 ± 6.80 pg mg^-1^ and 69.54 ± 6.76 pg mg^-1^, respectively. The presence of predator odour altered cortisol concentrations only at low structural complexity, resulting in a context-dependent physiological response.

**Table 2:** Results of two-way ANOVA on cortisol concentrations of juvenile *Pomacentrus chrysurus*.

Source	F	p	η^2^
Complexity _(2,56)_	1.14	0.327	0.039
Odour _(1,56)_	1.11	0.297	0.019
Complexity*Odour _(2,56)_	6.66	**0.003**	0.192

Summary of the effect of complexity and forewarning odours on cortisol concentrations from whole-body homogenate of juvenile *P*. *chrysurus*. Degree of freedom are presented with each model and bold values are significant at alpha = 0.05. Partial eta-squared (η^2^) are given as an effect size.

## Discussion

Predation risk can play an important part in predator-prey dynamics [10,13,73] and can also have cascading effects on community composition and ecosystem functions [8,48,74]. Our results show that a common coral reef damselfish (*P*. *chrysurus*) perceives risk based on available information and environmental context. Both the level of habitat complexity and olfactory predator cues can behaviorally modify escape responses in juvenile fishes, although we found the effects of complexity and olfactory cues on the behavioral response to be independent and not additive/interactive as hypothesized. Our results suggest that trends in the fast-start response may in part be associated with context-dependent cortisol concentrations and their influence on response motivation. The current study shows that the whitetail damselfish can modify anti-predation behaviours when presented with risk odours from a predator and similarly that increasing levels of complexity may be perceived as increased risk. However, our results are unable to ascertain the potential effect of the rearing environment on the phenotype of the escape responses [59,75], that is, being reared in high complexities environments may heighten escape responses. Clearly, this is an aspect that warrants further study.

Our results highlight the important role that environmental context plays in antipredation responses. In contrast to expectations, our results suggest that whitetail damselfish perceived increased complexity as increased risk, as their escape responses were heightened with increasing complexity. For example, between the low and high complexity treatments, the mean response speed of prey increased by 13.7% and the maximum speed increased by 13.3%. While we hypothesized that a lack of complexity would leave prey exposed and more vigilant, our data indicates fish reared in higher complexity environments had enhanced kinematic performance, potentially due to reduced visual information of the surrounding environment causing prey to over-estimate risk. Although shelter is known to provide many benefits to prey [52,55], complex habitats may challenge their ability to accurately assess risk and occasionally impede escape pathways [76]. The lack of visual information from increased turbidity has been shown to reduce exploration, activity levels and is associated with higher perception of predation risk in a freshwater minnow (*Pelasgus stymphalicus*) [73]. Similarly, Hess et al. [68], found that juvenile damselfish *Amphiprion melanopus* exposed to turbid environments displayed heightened escape response when compared to clear water controls. By reducing visual information, habitat complexity can increase the uncertainty of predation risk which can impact fitness. For instance, Rilov and colleagues [74] found that artificially reducing the field of view for the bicolor damselfishes (*Stegastes partitus*) decreased mating attempts and distance ventured from their nest. The low complexity environment in the current experiment offered more visual information, which is preferentially used by prey to gauge the intentions and motivation of any approaching threat [17], and therefore, may allow them to respond in an optimal manner.

Olfactory cues used in risk assessment provided forewarning and improved escape performance. In our study, juvenile whitetail damselfish perceived the predator odour of the rockcod to be a threat and responded by escaping faster. For example, when exposed to predator odours prior to the simulated predator strike, the mean response speed of prey increased by 14.9% compared to control treatment while exposure to odours of a non-threating herbivore had no significant impact on response speeds. These results support earlier findings on the whitetail damselfish [77], which showed juveniles appropriately assessed olfactory, visual, and combined cues of another rockcod (*C*. *microprion*) as a predation threat and enhanced both mean and maximum response speed in response, while exposure to a non-predatory butterflyfish (*Chaetodon trifasciatus*) had a substantially lower impact. Similarly, Ramasamy et al., [36] found that exposing damselfish (*Acanthochromis polyacanthus*) to olfactory, visual, and combined cues of a common predator (*Pseudochromis fucus*) improved escape responses. Together these results show that as levels of apparent predation risk increase, prey prime their escape performance to optimize energy use, which highlights the context-dependent nature of fishes’ escape response [33].

Although no interaction occurred between complexity and odours in the kinematic analysis, it is of interest to explore the relative impacts each treatment had on the escape response of *P*. *chrysurus*. When comparing the escape response between saltwater control and predator odours, the forewarning of a threat led to a more risk adverse response, escaping faster and further. However, when comparing the complexity treatments, we found that the escape responses of higher complexity treatment matched the magnitude that was induced by the predator odour treatment. Similarly, the intensity of escapes in low complexity treatments aligned with saltwater controls (See Fig 2). Using the partial eta-squared as an estimate of effect size we can see that complexity and odours had similar impacts on the escape variables (e.g. distance; complexity η^2^ = 0.054 and odour η^2^ = 0.063; See Table 1), indicating that prey perceived the rearing environment and lack of visual information to be roughly as threatening as olfactory cues of a known threat. Our data suggests that prey over-estimate the risk when visual information is restricted and that the potentially alleviating influence of shelter does not outweigh the uncertainty of predation risk in the whitetail damselfish.

A short-term rapid elevation in cortisol may be adaptive by increasing survival-related behaviors [41], since energy stores are utilized and redirected to improve cardiovascular, muscular, and cognitive abilities [43]. For example, exogenously increasing corticosterone levels in tree lizards (*Urosaurus ornatus*) and subsequently exposing them to a predator, enhanced their anti-predation behaviors [78]. Tree lizards with higher corticosterone levels had a faster latency to respond to the predator and hid longer than control lizards. In fishes, exogenous cortisol implants in the frillfin goby (*Bathygobius soporator*) were found to increase survival-related behaviours, such as reduced activity and increased sheltering [42]. Similarly, micropredation events by gnathiids have been shown to increase cortisol concentrations in the Ambon damselfish (*P*. *amboinensis*) and consequently, reduce activity levels [69]. However, this study found fast-start escape responses to be negatively impacted by increased cortisol. An explanation for the disparity between our results and those of Allan et al. [69] may be that the cortisol levels induced by gnathiids were much higher than in our study, suggesting that their stress response may represent a chronic effect which can become maladaptive [43]. The interaction effect between olfactory cues and habitat complexity on the cortisol response observed in the current study only partially aligns with the idea of improved survival behaviours. Our cortisol data indicates that fish from both predator odours and lower complexity treatments are stressed, suggesting their response may be similar and advantageous to survival. Although most kinematic fast-start escape variables were enhanced with the addition of predator odour, in the lower complexity treatments escape responses were unaffected as they aligned similarly with the saltwater controls. Our physiological results indicate that the structural environment can alleviate the stress of predation risk. However, to better understand the role that cortisol plays in modifying fast-start escape responses we suggest future studies explicitly test the effects of exogenously induced cortisol on the kinematic responses of fishes.

In addition to affecting behavioral responses, the threat of predation can also induce a stress response, however, environmental characteristics can interact with prey perception to mediate such responses. Woodley and Peterson [52] found that in the absence of shelter, the visual presence of a predator elevated cortisol levels by four-fold, and altered the metabolism and growth of the longnose killifish (*Fundulus majalis*). However, these effects were not observed when adequate shelter was provided [52]. Their study highlights both the impact predators can have on important hormonal, physiological, and whole-animal performance traits as well as the ability of shelter to mediate these impacts during predator-prey interactions. In the current experiment we found that predator odours and structural complexity interacted to influence cortisol concentrations in a context-dependent manner. The whitetail damselfish experienced higher cortisol levels when exposed to predator odours but only when structural complexity was low. In accordance with Woodley and Peterson [52], our data shows that the whitetail damselfish recognizes the threat of a predator odour, but only perceives it as a physiological stressor when shelter is lacking.

While our physiological data shows that the structural environment is important to mediate a stress response, a considerable amount of variability is present in our cortisol data, which may arise for a variety of reasons. Although 5 min has been shown to induce an acute stress response in the blackeye thicklip wrasse (*Hemigymnus melapterus*) from a physical stressor [67], the limited exposure time (5 min) of odours may have limited the rise in cortisol levels, considering we used a psychological or anticipatory stressor [38]. However, if too much time is provided between the odour and burst stimulus, prey may perceive no imminent threat. Additionally, although care was taken to transfer fish to arena tanks, this handling may have induced some stress and increased variability among all treatments. Likewise, allowing for additional acclimation time may have reduced this variability. Even with this variability, our finding of significant context-dependent physiological response between habitat complexity and threat of predation suggests the 5 min time period used was a sufficient timeframe for the current study, although longer may have reduced among-replicatevariance.

While many coral reef areas are transitioning from complex coral ecosystems to more homogenous algal-dominated seascapes, the impact of this degradation on predator-prey relationships is poorly understood. It has been suggested that as habitat complexity is lost, consumptive effects may increase [6], however, with reduced structural complexity comes the provision of more sensory information about potential threats. The current study suggests that higher levels of complexity may be perceived by prey to be higher risk and that the increased visual information in low complexity environments may help prey to appropriately assess risk and respond more effectively. With the capacity to better assess risk and modify escape responses, prey may be able to partly alleviate the risk of increased predator-prey interactions in more degraded environments. Additional work should attempt to understand how complexity may alter the rates of predator strikes and their success to provide a more complete picture of the ecology of predator-prey interactions in changing environments.

In conclusion, we found that the whitetail damselfish modified its fast-start escape response when forewarned with olfactory cues of a predator and that the degree of habitat complexity could additionally shape these responses. Higher habitat complexity was associated with improved escape performance, likely a result of overestimating risk by limiting visual information. Our whole-body cortisol levels suggested that whitetail damselfish became stressed when forewarned with predator odours, but only when complexity levels were low. The present study did not allow us to elucidate cortisol’s role in directly modifying anti-predator responses, and this represents an area for future studies.

## Supporting information

S1 Fig Overview of experimental tank. (DOCX)

S2 Fig Validation steps for ELISA cortisol analysis of whole-body homogenates of the white tail damselfish (*Pomacentrus chrysurus*). The optimal sample dilution (50% B/B_0_) was determined, and compared with the standard curve (A, dotted line). Based on the tested dilutions, an optimal dilution factor of 12 was used for analysing the remaining samples (A, dashed line). Second, parallelism was confirmed by comparing the slopes of the standard curve and the diluted samples (B). Third, the accuracy or extraction efficiency of cortisol from the fish samples was tested (C, means±SD, *n* = 4). The extraction efficiency (78.5%) was used as a correction factor for the samples.
(DOCX)

S1 Table Sample sizes for responsiveness of juvenile *Pomacentrus chrysurus* performing a C-start escape response compared by treatments, level of complexity and odour. (DOCX)

S2 Table Bonferroni corrected post-hoc comparisons for kinematic variables in Fig 2. Bold values are significant at alpha = 0.05.
(DOCX)

S3 Table Tukey’s HSD post-hoc comparisons for cortisol concentration presented in Fig 3. Bold values are significant at alpha = 0.05.
(DOCX)
