# Supplementary figures and images for: Habitat complexity and predator odours impact on the stress response and antipredation behaviour in coral reef fish
Source: PLoS One. 2023 Jun 28;18(6):e0286570. doi: 10.1371/journal.pone.0286570 (PMC10306203; doi:10.1371/journal.pone.0286570)

**Supporting information**

**Fig S1. Overview of experimental tank**


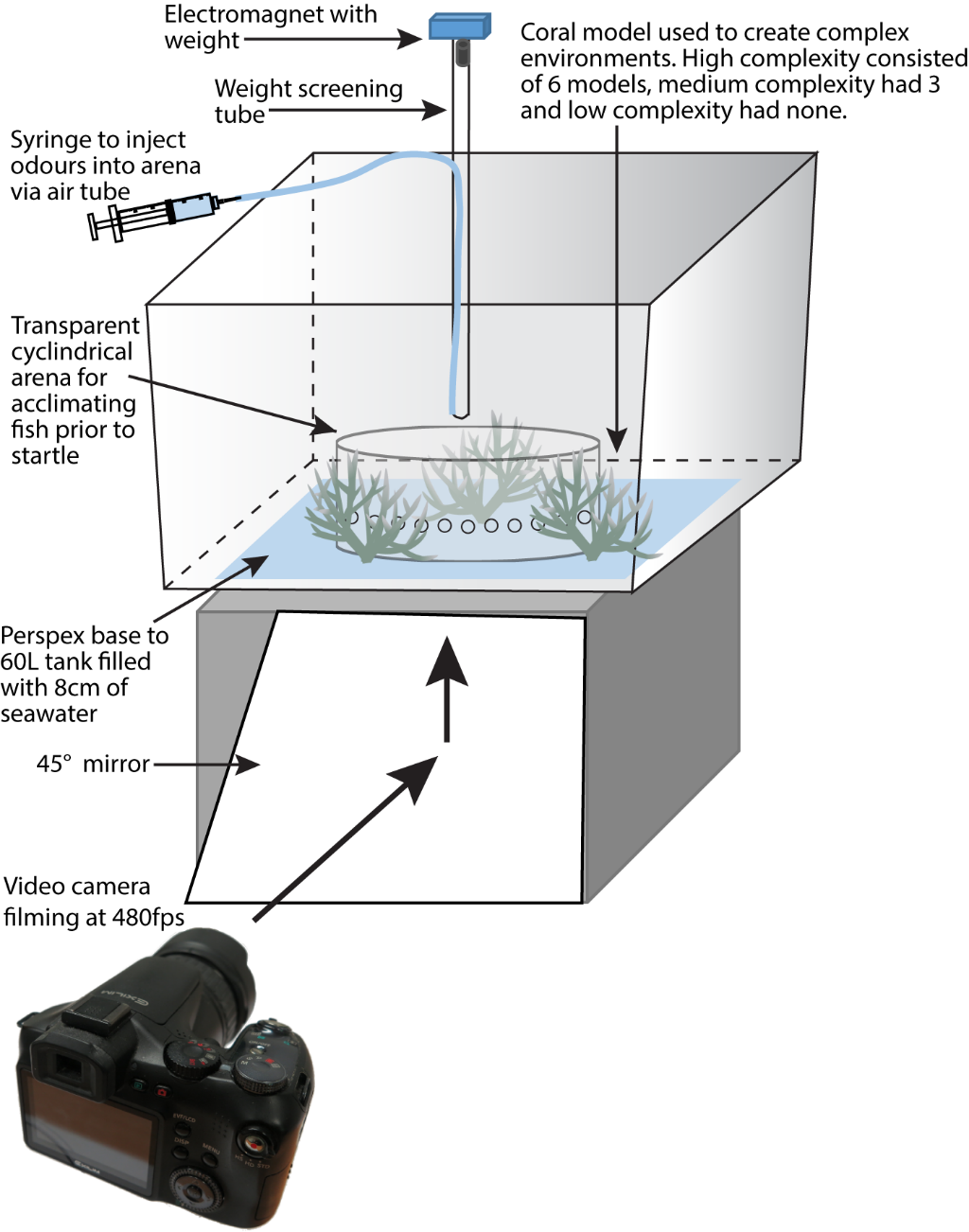

Supplement: S1 Fig — (DOCX) [file pone.0286570.s001.docx]
