# Supplementary material for: Habitat complexity and predator odours impact on the stress response and antipredation behaviour in coral reef fish
Source: PLoS One. 2023 Jun 28;18(6):e0286570. doi: 10.1371/journal.pone.0286570 (PMC10306203; doi:10.1371/journal.pone.0286570)
Supplement: S2 Fig — The optimal sample dilution (50% B/B0) was determined, and compared with the standard curve (A, dotted line). Based on the tested dilutions, an optimal dilution factor of 12 was used for analysing the remaining samples (A, dashed line). Second, parallelism was confirmed by comparing the slopes of the standard curve and the diluted samples (B). Third, the accuracy or extraction efficiency of cortisol from the fish samples was tested (C, means±SD, n = 4). The extraction efficiency (78.5%) was used as a correction factor for the samples. (DOCX) [file pone.0286570.s002.docx]

**Supporting information**

**Figure S2. Validation steps for ELISA cortisol analysis of whole-body homogenates of the white tail damselfish (*Pomacentrus chrysurus*).**


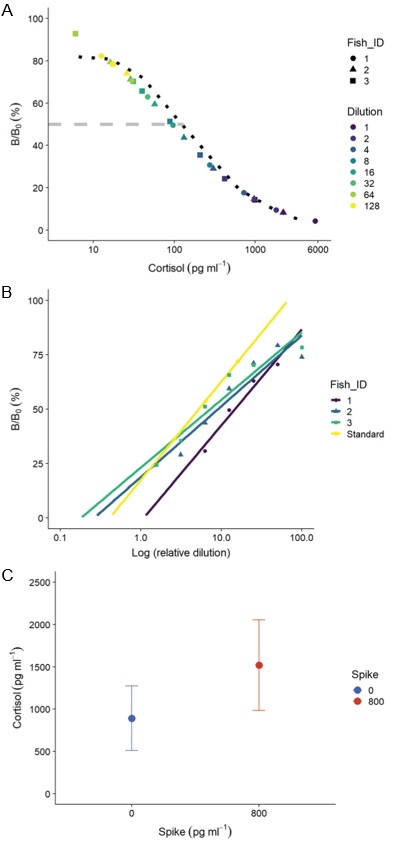


The optimal sample dilution (50 % B/B_0_) was determined, and compared with the standard curve (A, dotted line). Based on the tested dilutions, an optimal dilution factor of 12 was used for analysing the remaining samples (A, dashed line). Second, parallelism was confirmed by comparing the slopes of the standard curve and the diluted samples (B). Third, the accuracy or extraction efficiency of cortisol from the fish samples was tested (C, means±SD, *n* = 4). The extraction efficiency (78.5 %) was used as a correction factor for the samples.
