# Supplementary material for: Habitat complexity and predator odours impact on the stress response and antipredation behaviour in coral reef fish
Source: PLoS One. 2023 Jun 28;18(6):e0286570. doi: 10.1371/journal.pone.0286570 (PMC10306203; doi:10.1371/journal.pone.0286570)
Supplement: S1 Table — (DOCX) [file pone.0286570.s003.docx]

**Supporting information**

**Table S1** **Sample sizes for responsiveness of juvenile *Pomacentrus chrysurus* performing a C-start escape response compared by treatments, level of complexity and odour.**

| Complexity | Odour | Responsiveness | Non-responsiveness |
| --- | --- | --- | --- |
| Low | Control | 17 | 3 |
| Low | Herbivore | 13 | 7 |
| Low | Predator | 14 | 5 |
| Medium | Control | 17 | 3 |
| Medium | Herbivore | 18 | 3 |
| Medium | Predator | 14 | 7 |
| High | Control | 20 | 0 |
| High | Herbivore | 14 | 6 |
| High | Predator | 17 | 6 |
