# Supplementary material for: Habitat complexity and predator odours impact on the stress response and antipredation behaviour in coral reef fish
Source: PLoS One. 2023 Jun 28;18(6):e0286570. doi: 10.1371/journal.pone.0286570 (PMC10306203; doi:10.1371/journal.pone.0286570)
Supplement: S2 Table — Bold values are significant at alpha = 0.05. (DOCX) [file pone.0286570.s004.docx]

**Supporting information**

**Table S2** **Bonferroni corrected post-hoc comparisons for kinematic variables in figure 2**

| Variable | Treatment | Term (1) | Term (2) | Mean Diff (1-2) | Std. Error | Adj. P | LCI 95% | UCI 95% |  |
| --- | --- | --- | --- | --- | --- | --- | --- | --- | --- |
| Speed | Complexity | Low | Medium | -.076 | .035 | .090 | -.160 | .008 | |
|  |  |  | High | -.088 | .034 | **.033** | -.172 | -.005 | |
|  |  | Medium | Low | .076 | .035 | .090 | -.008 | .160 | |
|  |  |  | High | -.013 | .034 | 1.000 | -.094 | .069 | |
|  |  | High | Low | .088 | .034 | **.033** | .005 | .172 | |
|  |  |  | Medium | .013 | .034 | 1.000 | -.069 | .094 | |
|  | Odour | Predator | Herbivore | .040 | .035 | .788 | -.046 | .126 | |
|  |  |  | SW | .099 | .034 | **.012** | .017 | .180 | |
|  |  | Herbivore | Predator | -.040 | .035 | .788 | -.126 | .046 | |
|  |  |  | SW | .059 | .034 | .244 | -.022 | .140 | |
|  |  | SW | Predator | -.099 | .034 | **.012** | -.180 | -.017 | |
|  |  |  | Herbivore | -.059 | .034 | .244 | -.140 | .022 | |
|  |  |  |  |  |  |  |  |  | |
| Max. Speed | Complexity | Low | Medium | -.143 | .046 | **.007** | -.254 | -.031 | |
|  |  |  | High | -.123 | .046 | **.024** | -.233 | -.012 | |
|  |  | Medium | Low | .143 | .046 | **.007** | .031 | .254 | |
|  |  |  | High | .020 | .045 | 1.000 | -.088 | .129 | |
|  |  | High | Low | .123 | .046 | **.024** | .012 | .233 | |
|  |  |  | Medium | -.020 | .045 | 1.000 | -.129 | .088 | |
|  |  |  |  |  |  |  |  |  | |
| Max. | Complexity | Low | Medium | -.072 | .022 | **.005** | -.126 | -.018 | |
| Acceleration |  |  | High | -.042 | .022 | .182 | -.096 | .012 | |
|  |  | Medium | Low | .072 | .022 | **.005** | .018 | .126 | |
|  |  |  | High | .030 | .022 | .513 | -.023 | .083 | |
|  |  | High | Low | .042 | .022 | .182 | -.012 | .096 | |
|  |  |  | Medium | -.030 | .022 | .513 | -.083 | .023 | |
|  | Odour | Predator | Herbivore | .061 | .023 | **.026** | .005 | .116 | |
|  |  |  | SW | .001 | .022 | 1.000 | -.051 | .054 | |
|  |  | Herbivore | Predator | -.061 | .023 | **.026** | -.116 | -.005 | |
|  |  |  | SW | -.059 | .022 | **.021** | -.112 | -.007 | |
|  |  | SW | Predator | -.001 | .022 | 1.000 | -.054 | .051 | |
|  |  |  | Herbivore | .059 | .022 | **.021** | .007 | .112 | |
|  |  |  |  |  |  |  |  |  | |
| Distance | Complexity | Low | Medium | -.003 | .001 | .090 | -.007 | .000 | |
|  |  |  | High | -.004 | .001 | **.033** | -.007 | .000 | |
|  |  | Medium | Low | .003 | .001 | .090 | .000 | .007 | |
|  |  |  | High | -.001 | .001 | 1.000 | -.004 | .003 | |
|  |  | High | Low | .004 | .001 | **.033** | .000 | .007 | |
|  |  |  | Medium | .001 | .001 | 1.000 | -.003 | .004 | |
|  | Odour | Predator | Herbivore | .002 | .001 | .789 | -.002 | .005 | |
|  |  |  | SW | .004 | .001 | **.012** | .001 | .007 | |
|  |  | Herbivore | Predator | -.002 | .001 | .789 | -.005 | .002 | |
|  |  |  | SW | .002 | .001 | .245 | -.001 | .006 | |
|  |  | SW | Predator | -.004 | .001 | **.012** | -.007 | -.001 | |
|  |  |  | Herbivore | -.002 | .001 | .245 | -.006 | .001 | |

Bold values are significant at alpha = 0.05
