# Supplementary material for: Habitat complexity and predator odours impact on the stress response and antipredation behaviour in coral reef fish
Source: PLoS One. 2023 Jun 28;18(6):e0286570. doi: 10.1371/journal.pone.0286570 (PMC10306203; doi:10.1371/journal.pone.0286570)
Supplement: S3 Table — Bold values are significant at alpha = 0.05. (DOCX) [file pone.0286570.s005.docx]

**Supporting information**

**Table S3** **Tukey’s HSD post-hoc comparisons for cortisol concentration presented in figure 3**

| Term (1) | Term (2) | Mean Diff (1-2) | Std. Error | P | LCI 95% | UCI 95% |
| --- | --- | --- | --- | --- | --- | --- |
| Low / Control | High / Control | -5.174 | 9.590 | .994 | -33.472 | 23.124 |
|  | High / Predator | -9.170 | 9.120 | .914 | -36.080 | 17.740 |
|  | Low / Predator | -29.979 | 8.937 | **.017** | -56.349 | -3.608 |
|  | Medium / Control | -14.657 | 9.590 | .648 | -42.955 | 13.641 |
|  | Medium / Predator | 2.463 | 9.120 | 1.000 | -24.447 | 29.373 |
|  |  |  |  |  |  |  |
| Low / Predator | High / Control | 24.805 | 9.204 | .092 | -2.353 | 51.963 |
|  | High / Predator | 20.809 | 8.713 | .178 | -4.900 | 46.517 |
|  | Low / Control | 29.979 | 8.937 | **.017** | 3.608 | 56.349 |
|  | Medium / Control | 15.322 | 9.204 | .560 | -11.836 | 42.480 |
|  | Medium / Predator | 32.442 | 8.713 | **.006** | 6.734 | 58.151 |
|  |  |  |  |  |  |  |
| Medium / Control | High / Control | 9.483 | 9.840 | .927 | -19.550 | 38.516 |
|  | High / Predator | 5.487 | 9.382 | .992 | -22.195 | 33.169 |
|  | Low / Control | 14.657 | 9.590 | .648 | -13.641 | 42.955 |
|  | Low / Predator | -15.322 | 9.204 | .560 | -42.480 | 11.836 |
|  | Medium / Predator | 17.120 | 9.382 | .459 | -10.562 | 44.803 |
|  |  |  |  |  |  |  |
| Medium / Predator | High / Control | -7.638 | 9.382 | .964 | -35.320 | 20.045 |
|  | High / Predator | -11.634 | 8.900 | .780 | -37.895 | 14.628 |
|  | Low / Control | -2.463 | 9.120 | 1.000 | -29.373 | 24.447 |
|  | Low / Predator | -32.442 | 8.713 | **.006** | -58.151 | -6.734 |
|  | Medium / Control | -17.120 | 9.382 | .459 | -44.803 | 10.562 |
|  |  |  |  |  |  |  |
| High / Control | High / Predator | -3.996 | 9.382 | .998 | -31.678 | 23.686 |
|  | Low / Control | 5.174 | 9.590 | .994 | -23.124 | 33.472 |
|  | Low / Predator | -24.805 | 9.204 | .092 | -51.963 | 2.353 |
|  | Medium / Control | -9.483 | 9.840 | .927 | -38.516 | 19.550 |
|  | Medium / Predator | 7.638 | 9.382 | .964 | -20.045 | 35.320 |
|  |  |  |  |  |  |  |
| High / Predator | High / Control | 3.996 | 9.382 | .998 | -23.686 | 31.678 |
|  | Low / Control | 9.170 | 9.120 | .914 | -17.740 | 36.080 |
|  | Low / Predator | -20.809 | 8.713 | .178 | -46.517 | 4.900 |
|  | Medium / Control | -5.487 | 9.382 | .992 | -33.169 | 22.195 |
|  | Medium / Predator | 11.634 | 8.900 | .780 | -14.628 | 37.895 |

Bold values are significant at alpha = 0.05
